# Cardiovascular imaging following perioperative myocardial infarction/injury

**DOI:** 10.1038/s41598-022-08261-6

**Published:** 2022-03-15

**Authors:** Ketina Arslani, Danielle M. Gualandro, Christian Puelacher, Giovanna Lurati Buse, Andreas Lampart, Daniel Bolliger, David Schulthess, Noemi Glarner, Reka Hidvegi, Christoph Kindler, Steffen Blum, Francisco A. M. Cardozo, Bruno Caramelli, Lorenz Gürke, Thomas Wolff, Edin Mujagic, Stefan Schaeren, Daniel Rikli, Carlos A. Campos, Gregor Fahrni, Beat A. Kaufmann, Philip Haaf, Michael J. Zellweger, Christoph Kaiser, Stefan Osswald, Luzius A. Steiner, Christian Mueller, BASEL-PMI Investigators, Johanna Gueckel, Marcel Liffert, Gabriel Kanhouche, Lorraine Sazgary, Pai C. Yu, Alexandra Prepoudis, Samyut Shrestha, Pedro Lopez-Ayala, Michael Freese, Sandra Mitrovic, Katharina Rentsch, Angelika Hammerer-Lerchner

**Affiliations:** 1 Department of Cardiology and Cardiovascular Research Institute Basel (CRIB), University Hospital Basel, University of Basel, Basel, Switzerland; 2 Department of Internal Medicine, University Hospital Basel, University of Basel, Basel, Switzerland; 3 Unidade de Medicina Interdisciplinar em Cardiologia, Instituto do Coração, Hospital das Clínicas HCFMUSP, Faculdade de Medicina, Universidade de São Paulo, São Paulo, Brazil; 4 Department of Anaesthesiology, University Hospital Düsseldorf, Düsseldorf, Germany; 5 Department of Anaesthesiology, University Hospital Basel, University Basel, Basel, Switzerland; 6 Department of Anaesthesiology, Kantonsspital Aarau, Aarau, Switzerland; 7 Department of Vascular Surgery, University Hospital Basel, University of Basel, Basel, Switzerland; 8 Department of Spinal Surgery, University Hospital Basel, University of Basel, Basel, Switzerland; 9 Department Orthopedic Surgery, University Hospital Basel, University of Basel, Basel, Switzerland; 10 Department of Anaesthesiology, Kantonsspital St. Gallen, St. Gallen, Switzerland; 11 Department of Laboratory Medicine, University Hospital Basel, Basel, Switzerland; 12 Department of Laboratory Medicine, Kantonsspital Aarau, Aarau, Switzerland

**Keywords:** Diagnostic markers, Cardiology

## Abstract

Patients developing perioperative myocardial infarction/injury (PMI) have a high mortality. PMI work-up and therapy remain poorly defined. This prospective multicenter study included high-risk patients undergoing major non-cardiac surgery within a systematic PMI screening and clinical response program. The frequency of cardiovascular imaging during PMI work-up and its yield for possible type 1 myocardial infarction (T1MI) was assessed. Automated PMI detection triggered evaluation by the treating physician/cardiologist, who determined selection/timing of cardiovascular imaging. T1M1 was considered with the presence of a new wall motion abnormality within 30 days in transthoracic echocardiography (TTE), a new scar or ischemia within 90 days in myocardial perfusion imaging (MPI), and Ambrose-Type II or complex lesions within 7 days of PMI in coronary angiography (CA). In patients with PMI, 21% (268/1269) underwent at least one cardiac imaging modality. TTE was used in 13% (163/1269), MPI in 3% (37/1269), and CA in 5% (68/1269). Cardiology consultation was associated with higher use of cardiovascular imaging (27% versus 13%). Signs indicative of T1MI were found in 8% of TTE, 46% of MPI, and 63% of CA. Most patients with PMI did not undergo any cardiovascular imaging within their PMI work-up. If performed, MPI and CA showed high yield for signs indicative of T1MI.

**Trial registration:** https://clinicaltrials.gov/ct2/show/NCT02573532.

## Introduction

With over 300 million surgeries performed annually, perioperative complications are of major medical and economic relevance^1–4^. Perioperative myocardial infarction/injury (PMI) has recently been identified as the most common cardiac complication following non-cardiac surgery and is an important contributor to postoperative mortality^1–4^. PMI most often occurs with patients under anesthesia and high-dose analgesia. Therefore, more than 85% of patients with PMI do not experience typical ischemic symptoms^1–3,5,6^. In order to identify these patients at increased risk of death, systematic screening for PMI by measuring cardiac troponin pre- and postoperatively has been endorsed in patients with high cardiovascular risk^7–9^. Several different pathophysiological processes including type 1 myocardial infarction (T1MI)^7^ may lead to PMI. Therefore, detailed clinical assessment, 12-lead electrocardiogram (ECG), and cardiovascular imaging are required during PMI work-up for identification of the most likely underlying pathophysiology^1–3,10–14^. This is mandatory for the selection of the most appropriate therapy.

As PMI has only recently been identified as an important clinical entity^1–4^, the frequency of different cardiovascular imaging modalities applied after the detection of PMI within a clinical PMI screening program, and the impact of cardiology consultation on the selection of cardiovascular imaging modalities and their yield for possible T1MI are largely unknown. Therefore, we addressed these major uncertainties within a prospective international multicenter study.

## Methods

### Study design and population

This was a secondary analysis within the prospective multicenter observational study Basel-PMI (NCT02573532). The study was carried out according to the principles of the Declaration of Helsinki and was approved by the Ethics Committee of Northwestern Switzerland (EKNZ). Between September 2012 and September 2018, we included consecutive patients undergoing non-cardiac surgery at three hospitals (University Hospital Basel, Cantonal Hospital Aarau, both in Switzerland, and the Heart Institute (InCor), University Sao Paulo, Brazil) who were eligible for the routine institutional PMI screening and response program and provided written informed consent for registration in the BASEL-PMI database. We adhered to the STROBE reporting guidelines for observational studies (Supplement Table 1)^15^.

The screening and response system for PMI was part of the standard of care for high-risk patients undergoing non-cardiac surgery in the selected surgical departments at the participating hospitals. Patients were screened if they had a planned hospital stay exceeding 24 h after surgery and were considered at increased cardiovascular risk defined as being 65 to 85 years of age, OR 45 to 64 years of age with a history of coronary artery disease (CAD), peripheral arterial disease (PAD), or cerebrovascular disease. For this analysis, patients with PMI adjudicated due to a primarily extra-cardiac cause such as sepsis, cardiac trauma, or pulmonary embolism, or PMI due to heart failure and tachyarrhythmia, including atrial fibrillation were excluded (Supplemental Methods).

### Definition of PMI

Plasma concentrations of high-sensitivity cardiac troponin T (hs-cTnT assay [Elecsys, Roche diagnostics, Mannheim, Germany] in Basel and in Sao Paulo) and sensitive cardiac troponin I (s-cTnI assay [Siemens Dimension Vista; Siemens Health Care Diagnostics, Tarrytown, NY] in Aarau) were measured before surgery, and on postoperative days 1 and 2 as well as later if clinically indicated. Patients who did not have at least two measurements of the respective cTn assay were excluded. PMI was defined as an absolute increase from a preoperative value (or between two postoperative measurements if the preoperative measurement was missing) of ≥ 14 ng/L for hs-cTnT and ≥ 45 ng/L for s-cTnI (the respective 99^th^ percentile of both assays) within three days following surgery.

### PMI response and consultation by a cardiologist

On regular working days, PMI patients underwent a 12-lead ECG and clinical evaluation by the cardiologist on duty, unless patients were already under close supervision in the intensive care unit, where consultation by a cardiologist was not deemed necessary. Based on the evidence available at the time of the study, the presence of ST-segment elevation, ST-segment depression, or typical chest pain, and consequently, elevated troponin-levels were considered suggestive of T1MI and an indication for early coronary angiography following interdisciplinary discussion with the treating surgeon. The presence of perioperative hemodynamic instability with hypotension, tachyarrhythmia or severe postoperative anemia were considered suggestive of type 2 myocardial infarction and an indication for the correction of the respective trigger of myocardial supply–demand mismatch^1–3,5,6^. With the exception of patients who clearly fulfilled the criteria of the Universal definition of Myocardial Infarction for T1MI according to the ESC guidelines^7^, given the lack of evidence, no explicit guidance on the selection and/or timing of cardiac vascular imaging was provided by the cardiologist on duty.

### Cardiovascular imaging findings suggestive of T1MI

The cardiovascular imaging modalities were analyzed separately, as one individual patient may have undergone several tests.

#### Transthoracic echocardiography

A new regional wall motion abnormality was interpreted as suggestive of the occurrence of a possible T1MI^7,16^. The left ventricular ejection fraction (LVEF) was obtained using the modified Simpson's method, and valve dysfunction and wall motion abnormalities were prospectively recorded. Preoperative echocardiography up to one year before surgery and postoperative transthoracic echocardiography (TTE) up to 30 days after surgery were evaluated to assess the presence of a new regional wall motion abnormality (dyskinesia/akinesia/hypokinesia), which was documented if clearly detectable compared to the preoperative echocardiography. If a preoperative echocardiography was not available, a regional wall motion abnormality was assumed to be new in the absence of a history of myocardial infarction (MI) or other medical records indicating reduced LVEF.

#### Myocardial perfusion imaging

Using 99Tc-labelled single-photon emission computed tomography (SPECT) or ^82^Rb perfusion positron emission tomography (PET) up to 90 days after surgery, any new scar or new postoperative ischemia was interpreted as suggestive of the possible occurrence of T1MI^7,17^. The summed stress scores (SSS) quantified overall perfusion abnormality (scar plus ischemia), while the summed difference score (SDS) represented the extent of ischemia^18,19^. An SSS of 4 or greater was considered as abnormal (minimum of 5% of myocardium affected) and a reversible defect with an SDS of greater than 2 was defined as ischemia (minimum of 3% of myocardium is ischemic)^20^. In order to assess new scarring in the postoperative myocardial perfusion imaging (MPI), direct comparison with preoperative imaging up to one year before surgery was performed. If no preoperative MPI was available, a scar was assumed new in the absence of a history of MI or other medical records indicating reduced LVEF.

#### Coronary angiography

Using coronary angiography, the presence of Ambrose's type II lesion or a complex lesion as adjudicated by an experienced interventional cardiologist was interpreted as an indication of a T1MI^10^. Lesions with an obstruction of over 50% were documented. Each lesion was classified based on Ambrose’s classification^21,22^. According to this classification the lesions were divided into 4 types: Concentric (symmetric and smooth borders and a broad neck), type I eccentric (asymmetric stenosis with smooth narrowing), type II eccentric (asymmetric stenosis with a convex intraluminal obstruction and narrow neck due to overhanging edges or irregular borders, or both) and multiple irregularities (three or more serial, closely spaced narrowing or diffuse irregularities)^21–23^. The complexity of the lesions was also examined. Lesions with a stenosis of more than 50% and at least one of the following morphologies were identified as complex lesions^10,24^: intraluminal filling defect consistent with thrombus, plaque ulceration, plaque irregularity (haziness, defined by irregular margins or overhanging edges), impaired flow (TIMI flow < 3, except chronic total occlusion), Supplemental Figs. 1 and 2.

### Statistical analysis

The data are expressed as medians and interquartile range (IQR) for continuous variables and as numbers and percentages (%) for categorical variables, All variables were compared by the Mann Whitney U test for continuous variables and the Pearson chi-square or Fisher’s exact test for categorical variables, as appropriate.

Analyses were performed at imaging modality level and at patient level. For the patient level analysis, only the most invasive/sophisticated imaging modality per patient was considered (coronary angiography over MPI, MPI over echocardiography). All hypothesis testing was two-tailed, and P values less than 0.05 were considered to indicate statistical significance. Statistical analyses were performed using IBM SPSS Statistics for Mac, version 26.0 (IBM Corp., Armonk, NY) and R version 3.6.1 (R Foundation for Statistical Computing, Vienna, Austria).

## Results

**Figure 1:**
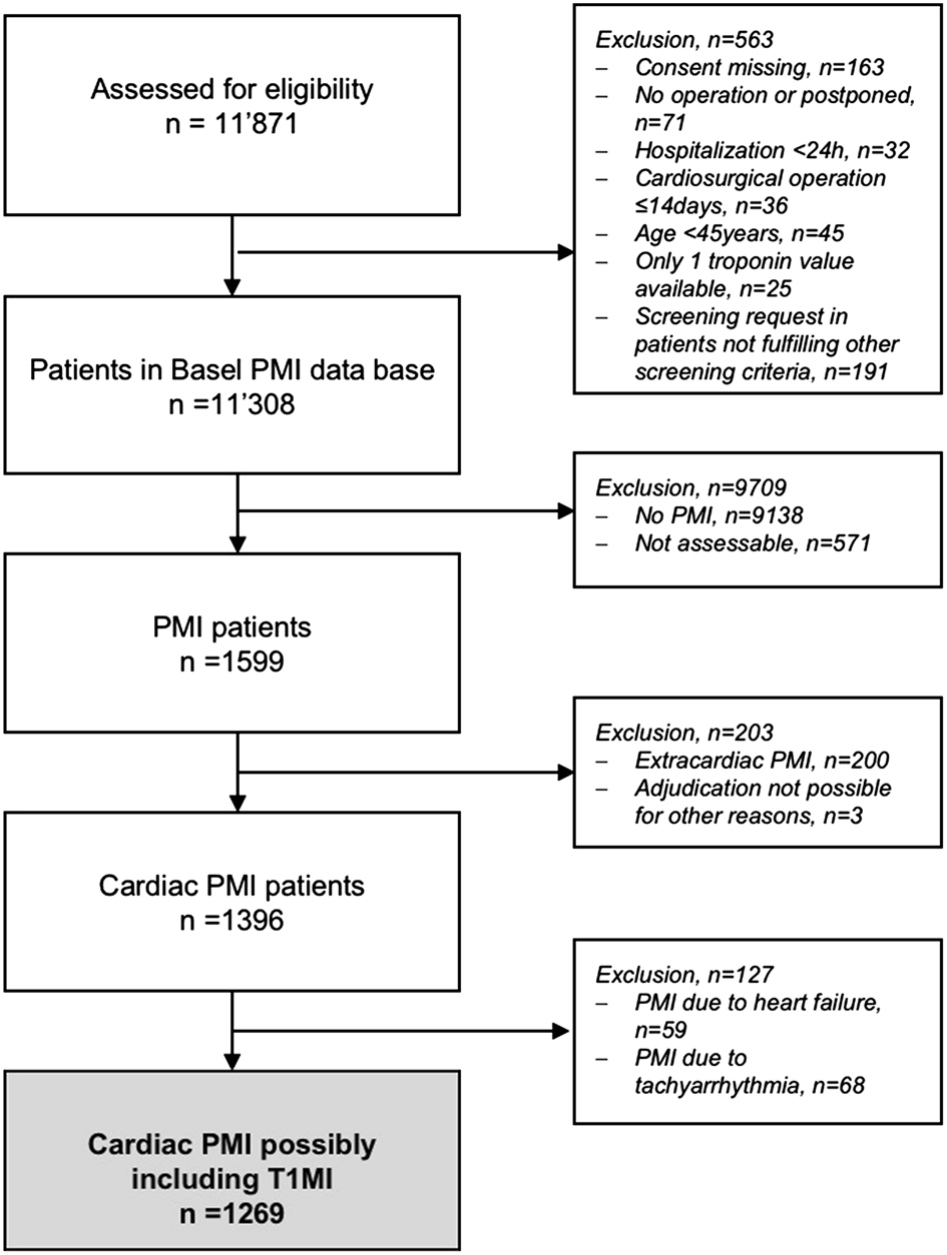
Patient flowchart showing number of patients assessed, number of patient excluded and reasons for exclusion. PMI: perioperative myocardial infarction/injury; T1MI: Type 1 myocardial infarction.

From 2012 until 2018, 11,871 consecutive patients were enrolled in the Basel-PMI study, of which 1599 patients developed a PMI. Among these patients, 1269 patients were eligible for this analysis (Fig. 1). The median age was 75 years, 39% (497/1269) were women, 45% (565/1269) had known CAD, 12% (156/1269) known stroke/TIA, 36% (459/1269) PAD, 30% (383/1269) diabetes mellitus, and 59% (755/1269) had chronic kidney disease (Table 1). Accordingly, 53% (661/1269) were on aspirin treatment and 56% (705/1269) on a statin at baseline.

**Table 1:** Baseline characteristics of patients with cardiac perioperative myocardial infarction/injury.

	All cardiac PMI n = 1269	With cardiology consultation n = 757	Without cardiology consultation n = 512	p-value
Age—median [IQR]	75 [69, 81]	75 [69, 80]	76 [70, 81]	0.003
Women—no. (%)	497 (39)	286 (38)	211 (41)	0.242
**Risk factors and history—no. (%)**				
Hypertension	947 (75)	583 (77)	364 (71)	0.021
**Diabetes**				0.339
NIDDM	203 (16)	130 (17)	73 (14)	
IDDM	180 (14)	103 (14)	77 (15)	
Coronary artery disease	565 (45)	340 (45)	225 (44)	0.777
History of myocardial infarction	281 (25)	152 (25)	129 (25)	0.895
Previous stroke/TIA	156 (12)	99 (13)	57 (11)	0.343
Atrial fibrillation	277 (25)	133 (22)	144 (28)	0.014
Chronic heart failure	245 (19)	150 (20)	95 (19)	0.627
Peripheral artery disease	459 (36)	331 (44)	128 (25)	< 0.001
COPD^¶^	221 (18)	102 (14)	119 (23)	< 0.001
Chronic kidney disease	755 (59)	430 (57)	325 (63)	0.020
**Preoperative medication—no. (%)**				
ASA	661 (53)	433 (59)	228 (45)	< 0.001
P2Y12 Inhibitors	112 (10)	59 (10)	53 (10)	0.964
β- Blocker	621 (49)	390 (52)	231 (45)	0.029
Statin	705 (56)	454 (60)	251 (49)	< 0.001
ACEI/ARB	589 (46)	366 (48)	223 (44)	0.105
**Surgery—no. (%)**				< 0.001
Orthopedic/traumatology	382 (30)	233 (31)	149 (29)	
Visceral	104 (8)	44 (6)	60 (12)	
Vascular	379 (30)	275 (36)	104 (20)	
Thoracic	114 (9)	46 (6)	68 (13)	
Spinal	162 (13)	97 (13)	65 (13)	
Urologic	102 (8)	49 (6)	53 (10)	
Other	26 (2)	13 (2)	13 (3)	
**ESC/ESA risk—no. (%)**				0.947
Low	230 (18)	135 (18)	95 (19)	
Medium	778 (61)	466 (62)	312 (61)	
High	261 (21)	156 (21)	105 (21)	
**Revised cardiac risk score—no. (%)**				0.346
I	331 (26)	203 (27)	128 (25)	
II	424 (33)	239 (32)	185 (36)	
III	290 (23)	174 (23)	116 (23)	
IV	224 (18)	141 (19)	83 (16)	
**Standard evaluation for PMI—no. (%)**				
Ischemic symptoms	136 (12)	102 (16)	34 (7)	< 0.001
Ischemic ECG findings	180 (14)	153 (26)	27 (18)	0.045
**Peak troponin—median [IQR]**				
S-cTnI	154.0 [97, 429]	179.5 [107, 730]	120.0 [81, 227]	0.004
Hs-cTnT troponin T	59.0 [40, 109]	61.0 [41, 124]	56.0 [38, 93]	0.001

Hs-cTnT was only measured in patients from the University Hospital Basel, Switzerland and INCOR Heart institute, Sao Paulo, Brazil; S-cTnI was only measured in patients from the Cantonal Hospital Aarau, Switzerland;

*PMI* perioperative myocardial infarction/injury, *IQR* interquartile range, *NIDDM* non-insulin-dependent diabetes mellitus, *IDDM* insulin-dependent diabetes mellitus, *TIA* transient ischemic attack, *COPD* chronic obstructive pulmonary disease, *ASA* acetylsalicylic acid, *ACEI/ARB* angiotensin-converting enzyme inhibitor/ angiotensin receptor blocker, *ESC/ESA* European Society of Cardiology/European Society of Anesthesiology, *S-cTnI* sensitive cardiac troponin I, *Hs-cTnT* high-sensitivity cardiac troponin T.

### PMI characteristics

Postoperatively, the median peak hs-cTnT concentration was 59 ng/L (IQR 40, 109), the median peak s-cTnI concentration was 154 ng/L (IQR 97, 429), 12% (136/1269) of patients had ischemic symptoms, and 14% (180/1269) presented ischemic ECG changes.

### Cardiology consultation and cardiovascular imaging

Most PMI patients (60%, 757/1269) were consulted by a cardiologist. Patients receiving cardiology consultation had higher peak cTn concentrations, and more often presented ischemic symptoms and/or ECG findings compared to patients without cardiology consultation.

**Figure 2:**
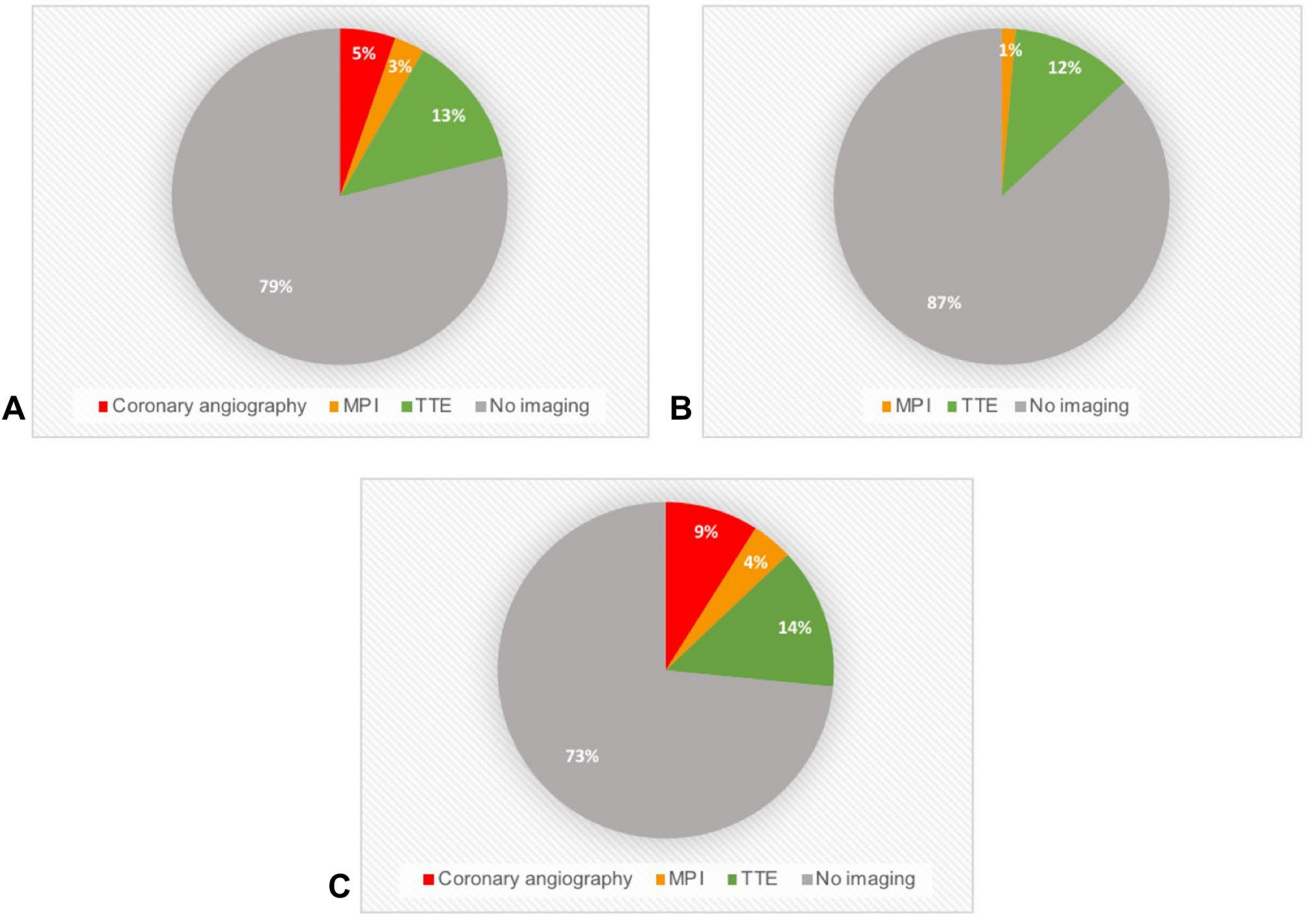
Patient level analysis: Cardiac imaging modalities in cardiac PMI. (**A**) All patients. (**B**) Patients with no cardiology consultation. (**C**) Patients with cardiology consultation. Pie chart showing the frequency of cardiac imaging modalities in general and in patients with and without cardiology consultation. MPI: myocardial perfusion imaging. TTE: transthoracic echocardiography.

Overall, 21% (268/1269) of patients with PMI underwent at least one cardiovascular imaging modality. Echocardiography was performed in 13% (163/1269) of patients within 30 days, MPI in 3% (37/1269) within 90 days and coronary angiography in 5% (68/1269) of patients within 7 days after surgery (Fig. 2A). Echocardiography had been used as the first cardiac imaging modality in 11/37 patients undergoing MPI and in 8/68 patients undergoing coronary angiography. Patients consulted by a cardiologist more often underwent cardiovascular imaging (27% versus 13%, p < 0.001) versus patients without cardiology consultation (Fig. 2B,C).

### Cardiovascular imaging evidence suggestive of T1MI

#### Echocardiography

**Figure 3:**
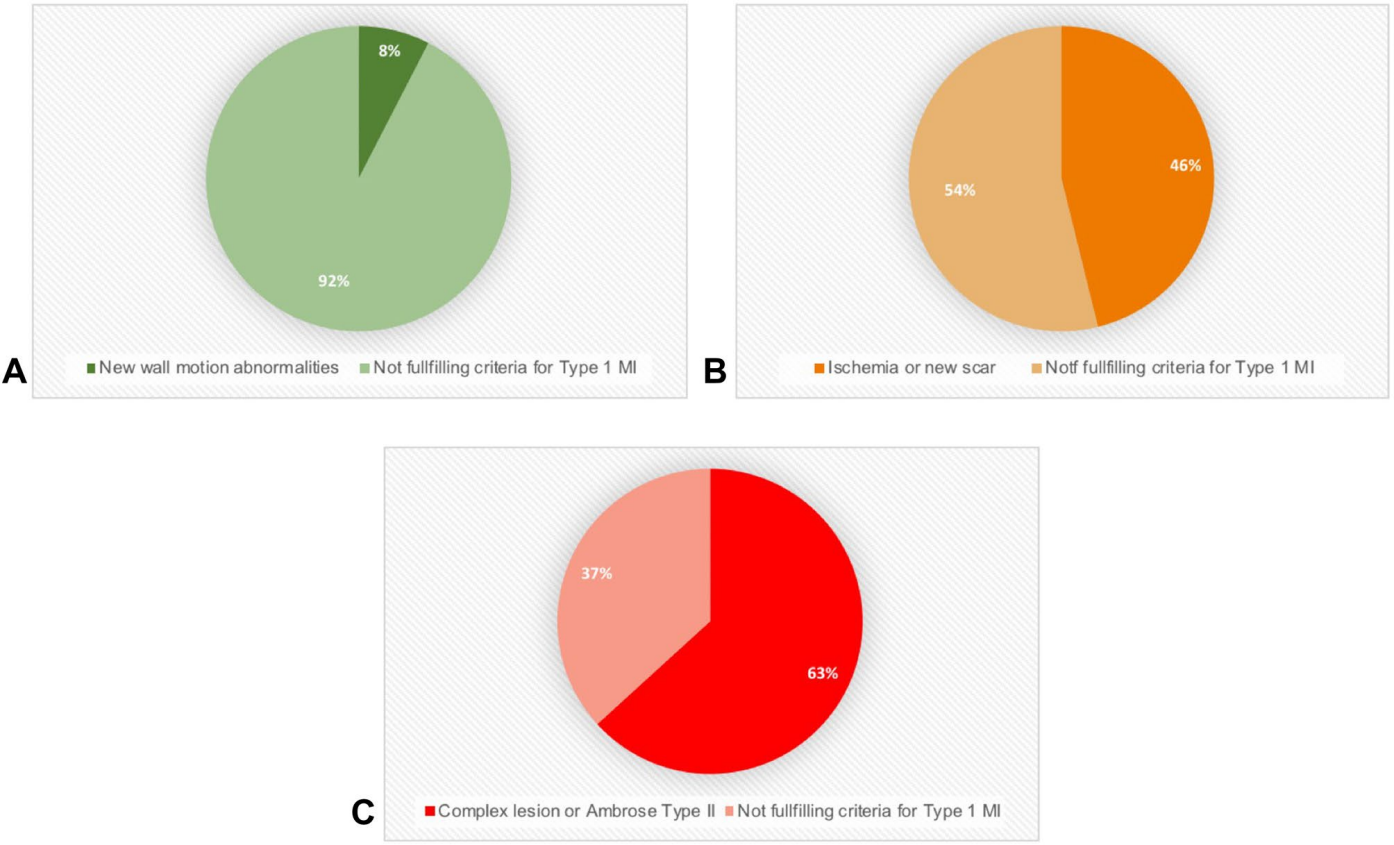
Imaging modality level analysis. (**A**) Transthoracic echocardiography. (**B**) Myocardial perfusion imaging. (**C**) Coronary angiography. Pie chart showing the yield for signs of type 1 MI in different cardiac imaging modalities. MI: Myocardial infarction.

Among 198 TTE, a new wall motion abnormality was identified in 15 (8%). These patients had significantly higher hs-cTnT concentrations (median 240 ng/L, IQR [109–504] vs. 85 ng/L, IQR [49–162], p = 0.005) and a significantly lower postoperative LVEF (median 45%, IQR [39–55%] vs. 55% IQR [45–60%], p = 0.043) than patients without a new wall motion abnormality (Table 2 and Fig. 3A).

**Table 2:** Evidence suggestive of type 1 myocardial infarction in different cardiac imaging modalities.

	Coronary angiography n = 68			Myocardial perfusion imaging/positron emission tomography n = 39			Transthoracic echocardiography n = 198		
	T1MI criteria present (n = 43)	T1MI criteria absent (n = 25)	p-value	T1MI criteria present (n = 18)	T1MI criteria absent (n = 21)	p-value	T1MI criteria present (n = 15)	T1MI criteria absent (n = 184)	p-value
Age—median [IQR]	74.0 [70.5, 77.5]	71.0 [68.0, 74.0]	0.056	72.5 [71.0, 74.8]	72.0 [69.0, 79.0]	0.366	79.0 [74.5, 82.5]	76.0 [70.0, 81.0]	0.236
Female gender—no. (%)	9 (21)	8 (32)	0.468	5 (28)	5 (24)	1.000	7 (47)	67 (36)	0.608
**Risk factors and history—no. (%)**									
Hypertension	33 (77)	19 (76)	1.000	16 (89)	18 (86)	1.000	9 (60)	137 (74)	0.361
Diabetes	17 (39)	8 (30)	0.605	9 (50)	7 (33)	0.615			0.553
NIDDM	11 (26)	5 (20)		5 (28)	4 (19)		1 (7)	27 (1 5)	
IDDM	6 (14)	2 (8)		4 (22)	3 (14)		4 (27)	33 (18)	
Coronary artery disease	26 (60)	13 (52)	0.670	10 (56)	8 (38)	0.442	6 (40)	90 (49)	0.692
History of myocardial infarction	10 (28)	8 (38)	0.608	5 (28)	6 (29)	1.000	1 (7)	53 (29)	0.074
Previous stroke/TIA	8 (19)	2 (8)	0.304	1 (6)	5 (24)	0.190	0 (0)	21 (11)	0.376
Atrial fibrillation	4 (11)	2 (10)	1.000	4 (22)	4 (19)	1.000	3 (20)	41 (22)	1.000
Chronic heart failure	9 (21)	4 (16)	0.754	6 (33)	1 (5)	0.035	3 (20)	47 (26)	0.765
Peripheral artery disease	23 (53)	12 (48)	0.853	7 (39)	5 (24)	0.488	6 (40)	58 (32)	0.698
COPD	4 (9)	3 (12)	0.702	3 (17)	1 (5)	0.318	2 (13)	32 (17)	1.000
Renal failure	23 (53)	12 (48)	0.853	12 (67)	13 (62)	1.000	9 (60)	115 (62)	1.000
**Preoperative medication—no. (%)**									
ASA	30 (75)	17 (68)	0.742	9 (50)	12 (57)	0.901	8 (53)	95 (52)	1.000
P2Y12 inhibitors	2 (7)	2 (9)	1.000	1 (6)	2 (10)	1.000	1 (7)	15 (8)	1.000
β- Blocker	27 (63)	12 (48)	0.350	8 (44)	9 (43)	1.000	8 (53)	84 (46)	0.761
Statin	23 (53)	15 (60)	0.789	11 (61)	9 (43)	0.415	4 (27)	90 (49)	0.113
ACEI/ARB	18 (42)	12 (48)	0.812	15 (83)	11 (52)	0.051	7 (47)	85 (46)	1.000
**Surgery—no. (%)**			0.304			0.403			0.284
Orthopedic/traumatology	12 (28)	5 (20)		5 (28)	5 (24)		4 (27)	72 (39)	
Visceral	0 (0)	2 (8)		NA	NA		4 (27)	13 (7)	
Vascular	24 (5)	10 (40)		4 (22)	3 (14)		4 (27)	42 (23)	
Thoracic	2 (5)	2 (8)		1 (6)	3 (14)		1 (7)	15 (8)	
Spinal	3 (7)	3 (12)		7 (39)	4 (19)		2 (13)	24 (13)	
Urologic	1 (2)	2 (8)		1 (6)	5 (24)		0 (0)	15 (8)	
Other	1 (2)	1 (4)		0 (0)	1 (5)		0 (0)	3 (2)	
**ESC/ESA risk—no. (%)**			0.892			0.908			0.389
Low	7 (16)	5 (20)		4 (22)	6 (29)		1 (7)	39 (21)	
Medium	23 (53)	12 (48)		11 (61)	12 (57)		10 (67)	111 (60)	
High	13 (30)	8 (32)		3 (17)	3 (14)		4 (27)	34 (18)	
**Revised cardiac risk score—no. (%)**			0.639			0.907			0.682
I	8 (19)	8 (32)		4 (22)	7 (33)		5 (33)	46 (25)	
II	12 (28)	7 (28)		6 (33)	6 (29)		4 (27)	64 (35)	
III	14 (33)	6 (24)		6 (33)	6 (29)		2 (13)	40 (22)	
IV	9 (21)	4 (16)		2 (11)	2 (10)		4 (27)	34 (18)	
**Standard evaluation for PMI—no. (%)**									
Ischemic symptoms	14 (33)	9 (36)	0.981	4 (22)	3 (14)	0.682	4 (27)	36 (20)	0.508
Ischemic ECG findings	26 (60)	8 (33)	0.061	3 (18)	3 (15)	1.000	5 (42)	45 (31)	0.521
**Peak troponin—median (IQR)**									
S-cTnI	745.0 [192, 2001]	539.5 [125, 1262]	0.561	NA	NA		NA	183.0 [127.0, 803.0]	NA
Hs-cTnT troponin T	238.5 [108, 738]	124.0 [86, 518]	0.299	108.5 [77, 201]	66.0 [46, 137]	0.071	240.0 [109, 504]	85.0 [49, 162]	0.003

Hs-cTnT was only measured in patients from the University Hospital Basel, Switzerland and INCOR Heart institute, Sao Paulo, Brazil; S-cTnI was only measured in patients from the Cantonal Hospital Aarau, Switzerland;

*T1MI* type 1 myocardial infarction, *IQR* interquartile range, *NIDDM* non-insulin-dependent diabetes mellitus, *IDDM* insulin-dependent diabetes mellitus, *TIA* transient ischemic attack, *COPD* chronic obstructive pulmonary disease, *ASA* acetylsalicylic acid, *ACEI/ARB* angiotensin-converting enzyme inhibitor/angiotensin receptor blocker, *ESC/ESA* European Society of Cardiology/European Society of Anesthesiology, *S-cTnI* sensitive cardiac troponin I, *Hs-cTnT* high-sensitivity cardiac troponin T, *NA* not available.

#### Myocardial perfusion imaging

Among 39 myocardial perfusion scans, 18 (46%) had evidence of either a new scar or ischemia or both (median SDS 6 [IQR 5–9.5], median SSS 11 [IQR 8–12]). These patients had higher hs-cTnT concentrations and more often a history of heart failure versus patients without scar/ischemia (Table 2 and Fig. 3B).

#### Coronary angiography

**Figure 4:**
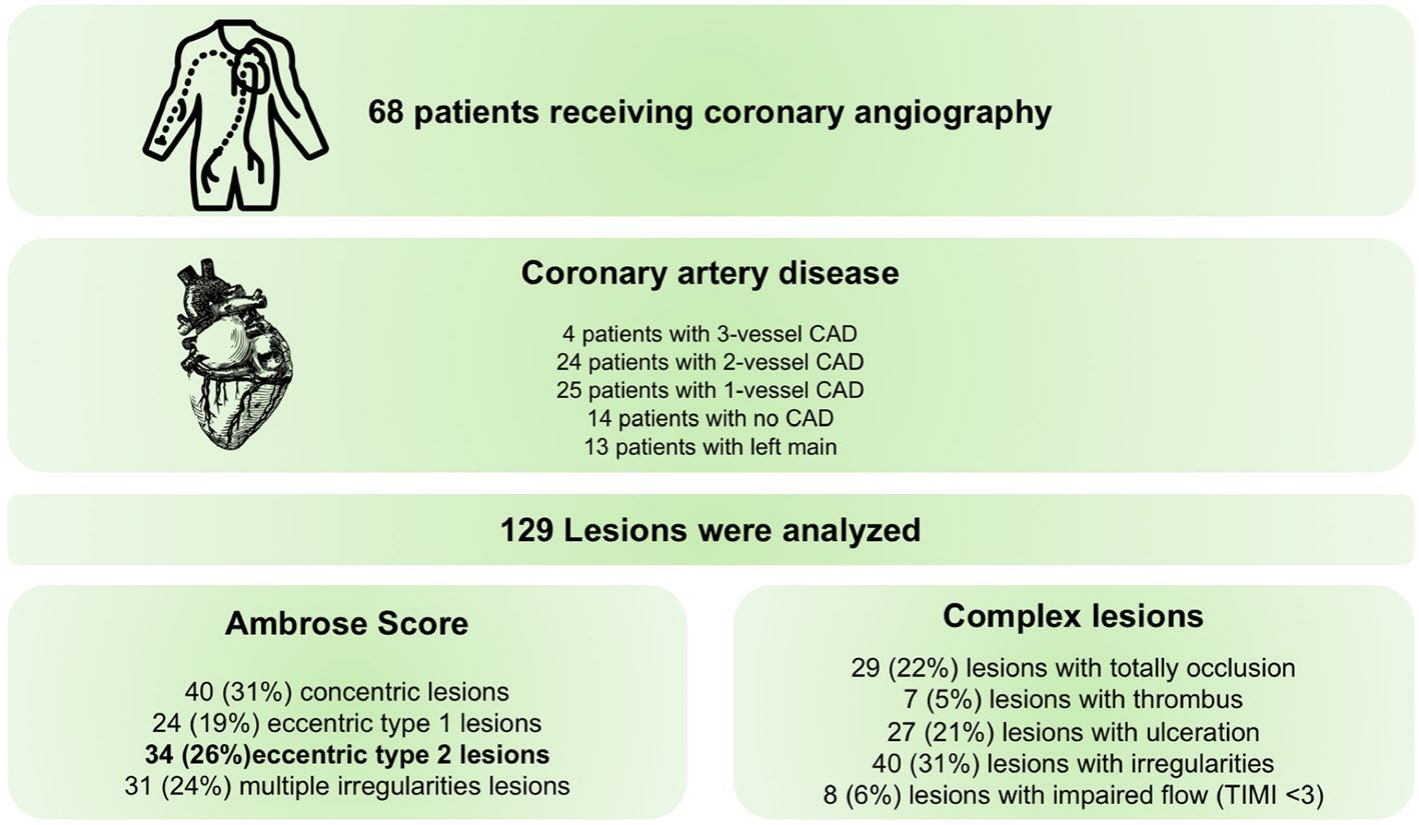
Details and summary of information collected from coronary angiography records. CAD: coronary artery disease. TIMI: Thrombolysis in Myocardial Infarction.

Among 68 coronary angiographies, 43 (63%) presented Ambrose's type II or complex lesions (Fig. 3C). Overall, 28 (41%) showed multi-vessel-disease, 13 (19%) significant stenosis of the left main vessel, and in 14 (21%) there was no evidence of significant CAD (Fig. 4). The median time from surgery to angiography was 4 days (IQR 2–6) and the median time from detection of PMI to angiography was 2 days (IQR 1–4). Myocardial revascularization was performed in 33 patients (47%). After a positive MPI, 3 (8%) patients underwent coronary angiography within 7 days and 8 patients within 90 days postoperatively.

## Discussion

This large, international, multicenter study prospectively included high-risk patients undergoing non-cardiac surgery in a PMI-screening program. We evaluated the frequency of cardiovascular imaging and its yield for findings suggestive of the possible occurrence of T1MI, the PMI phenotype with the best documented therapeutic consequences^10,11,13,25,26^. We report five major findings.

First, patients developing PMI were older (median age 75 years) with extensive pre-existing cardiovascular comorbidity, including CAD in 45%, PAD in 36%, atrial fibrillation in 25%, diabetes mellitus in 30% and chronic kidney disease in 59%^27,28^. Second, despite the well documented association between PMI and death within 30-days following non-cardiac surgery^1–3,10–14^, the vast majority (79%) of patients in whom PMI was detected did not undergo any cardiovascular imaging postoperatively. Third, cardiac consultation and large PMI size as quantified by peak cTn seemed to modify the frequency of cardiovascular imaging being performed. Fourth, echocardiography was the cardiovascular imaging modality most commonly used and was performed in 13% of patients with PMI, followed by coronary angiography in 5% and MPI in 3%. Fifth, when performed as part of the PMI work-up, the incidence of findings suggestive of the possible occurrence of T1MI varied widely among the three imaging modalities. The incidence of new wall motion abnormalities was low with echocardiography (8%) and was moderate-to-high for the presence of a new scar/ischemia with MPI (46%), and high for Ambrose's type II or complex lesions (63%) with coronary angiography.

These findings extend and corroborate prior studies on the detection and management of PMI, a silent and neglected killer following non-cardiac surgery^1–3,5,6^. PMI predominately affects patients burdened by four factors: older age, high prevalence of end organ damage from atherosclerosis including CAD, stroke, and PAD, as well as chronic kidney disease, wounds, and impaired mobility following major surgery, and the often-severe underlying non-cardiac disorder that required major surgery. These complexities substantially increase the challenges associated with early diagnosis of T1MI. Simple and more readily available tools such as ECG only play a supportive role, as the minority of patients with PMI (< 15% in patients adjudicated to cardiac PMI) have distinctive ischemic ECG changes^29^. In addition, these complexities also increase the risk of complications associated with cardiovascular imaging modalities, particularly those using contrast agents, but likely decrease the likelihood of benefit from therapeutic interventions specific to T1MI on health-related quality of life and long-term mortality. These considerations may explain at least in part the very low use of cardiovascular imaging following PMI. Underestimation of the substantial risk of 30-day mortality associated with PMI (about 10%) among the treating clinicians as well as the cardiologist on duty may also have played a role^1–3,12^. On the other hand, early diagnosis of T1MI is essential for early initiation for evidence-based and ultimately potentially life-saving therapy^16^.

Predicting PMI is challenging. Although it is well known that patients with known heart disease or with a high burden of risk factors are at risk for developing PMI, we recently demonstrated that procedure-related factors, such as high risk surgery according to the Revised Cardiac Risk Index, surgery duration and bleeding play a larger role in the occurrence of PMI than patient-related risk factors^30^. Therefore, systematic screening for prompt detection of a PMI and the implementation of adequate actions gains further importance. After detecting the PMI, it is essential to determine its probable etiology, since proper cardiac work-up depends on the cause^12^. As a screening tool to diagnose PMI, some guidelines propose surveillance with postoperative ECG and cardiac troponins in the first 2–3 days after surgery^9^. This systematic assessment of different cardiovascular imaging modalities following PMI detection within a systematic PMI-screening and clinical response program may help institutions to estimate the imaging workload and costs associated with the implementation of a PMI screening program. It also extends and complements important single-center pilot studies of patients identified clinically with PMI undergoing coronary angiography due to suspected T1MI^10,11,13^. Among 120 patients enrolled in Sao Paolo, 45% had Ambrose’s type 2 lesions versus 57% in a control cohort with spontaneous MI^10^. Among 66 patients enrolled in New York, 26% had evidence of a thrombotic process suggesting T1MI^11^. Among 30 patients enrolled in Hamilton, 13% presented evidence of thrombus on optical coherence tomography versus 67% in a control cohort with spontaneous MI^13^.

The different frequencies of the cardiovascular imaging modalities investigated seemed to mainly reflect their different availability and ease of use. Appropriate clinical selection of the cardiovascular imaging modalities to be used was further documented by the increasing yield regarding the possible occurrence of T1MI with increasing invasiveness and cost of the procedures. Given the much higher sensitivity for detecting ischemic myocardium versus echocardiography, MPI seemed to have been underused in this study.

Some limitations warrant consideration when interpreting our findings. First, as it is a retrospective assessment of cardiovascular imaging performed according to the clinical decision of the treating physician, referral bias rather than method-inherent characteristics should be considered the main determinant for the different yields for the signs of T1MI observed, as only a subgroup of patients (only patients referred for any type of cardiovascular imaging by the attending cardiologist) were examined. Additionally, no comparisons between patients with and without cardiovascular imaging were performed. Second, while clinically well-established and useful, all the cardiovascular imaging techniques investigated have inherent limitations regarding the detection of acute atherothrombosis as the pathophysiological signature underlying T1MI. However, lesions with complex morphology and Ambrose’s type II lesions were found to be strongly correlated with plaque rupture^10,31,32^. Moreover, the 4th universal definition of MI lists new wall motion abnormalities and loss of viable myocardium in conjunction with an increase in cTn^7^ as definite criteria associated with T1MI. Third, the average time from PMI to angiography was 2 days (IQR 1,4). During this time possible thrombus formation with autolysis could have occurred, possibly leading to an underestimation of T1MI signs.

In conclusion, only about one out of five patients developing PMI underwent cardiovascular imaging for a PMI work-up. If performed, MPI and CA present a high yield for signs indicative of the possible occurrence of T1MI.

## Supplementary Information

Supplementary Information.

## Data Availability

Based on the BASEC Ethics Approval, the raw data cannot be shared publicly. However, anonymized data that underlie the results reported in this article will become available to all interested parties, after the publication. Requests can be made to the corresponding author. Data requestors will need to sign a data access agreement.
