# Supplementary material for: Cardiovascular imaging following perioperative myocardial infarction/injury
Source: Sci Rep. 2022 Mar 15;12:4447. doi: 10.1038/s41598-022-08261-6 (PMC8924205; doi:10.1038/s41598-022-08261-6)
Supplement: Supplementary file 1 — Supplementary Information. [file 41598_2022_8261_MOESM1_ESM.docx]

**Supplement Material**

# Supplemental Methods

## Clinical PMI Screening

Screening was implemented for patients undergoing visceral, orthopaedic, trauma, vascular, urologic, spinal, and thoracic surgical procedures. To improve compliance with the screening program, clinicians were alerted automatically of eligibility to the program based on the electronic health records. Serial high-sensitivity cardiac troponin T (hs-cTnT) or sensitive cardiac troponin I (s-cTnI) measurements were ordered by the treating anaesthesiologist. Patients underwent cTn monitoring and were registered into the database multiple times if a minimum of 5 days had elapsed between procedures.

## Rational for definition of PMI

Based on findings from prior studies showing that asymptomatic elevations in cTn were also associated with increased short-term mortality(1), we chose to not mandate specific symptoms or specific ECG changes into the definition of PMI. We used delta values instead of maximum postoperative levels to ensure that our definition reflected “acute” myocardial damage and was time-related to surgery, thus avoiding misclassification of chronically elevated levels. Chronic hs-cTn elevations are expected in a relevant amount of (surgical) patients(2), and were previously shown to be independently associated with increased risk of death and major adverse cardiac events(3). We chose an absolute rather than a relative delta cTn level for the diagnosis of PMI, because absolute changes have shown higher diagnostic accuracy as compared to relative changes in the detection of acute MI in the non-operative setting(4,5). The absolute increase of ≥14ng/L was selected as 14ng/L represents the 99^th^ percentile of healthy individuals and thereby all PMIs invariably would fulfill the change as well as the absolute cTn criteria of the universal definition(6). In analogy a cut-off of 45ng/L was chosen for s-cTnI, as it represents the 99^th^ percentile for this assay.

## cTn measurements

Hs-cTnT was measured using an Cobas Elecsys (Roche Diagnostics) assay with limit of detection (LoD) of 5ng/L, a 10% coefficient of variation at 13ng/L and the 99^th^ percentile of a healthy reference population at 14ng/L(7).

S-cTnI was measured using a Siemens Dimension Vista assay (Siemens Health Care Diagnostics, Tarrytown, NY). This s-cTnI assay has a LoD of 15ng/L and a 99^th^ percentile 45ng/L.

## Clinical data collection

### Baseline variable definitions

Coronary artery disease was defined as history of coronary artery disease, history of acute myocardial infarction, finding of stenosis on coronary angiogram, or positive stress testing.

Peripheral artery disease was defined as history of peripheral artery disease, known carotid stenosis, or arterial vascular surgery for aortic aneurysm.

Stroke was defined as history of acute new focal neurological deficit judged by treating physicians to be of vascular cause lasting >24 hours

Chronic heart failure was defined as history of congestive heart failure, left ventricular ejection fraction ≤40%, or diastolic dysfunction grade II or higher with elevated B-type natriuretic peptide irrespective of ejection fraction.

Atrial fibrillation was defined as history of at least paroxysmal atrial fibrillation occurring more than once, or atrial fibrillation on preoperative ECG.

Chronic kidney disease was defined either as known chronic kidney disease or using preoperative creatinine values and estimated glomerular filtration rate (eGFR) calculated using the CKD-EPI formula. An eGFR of <60ml/min was considered as evidence of chronic renal disease.

### Perioperative variables

Bleeding was defined as a drop of haemoglobin >30g/L from pre- to postoperative values or need for ≥1 red blood cell transfusion during or within the first two days after surgery. Bleeding as seen as intraoperative if the drop in haemoglobin occurred on the day of surgery, and postoperative if from day 1-2.

## Adjudication criteria

**PMI adjudication**

After 1-year follow-up, the etiology of all PMI was adjudicated by two independent experts based on all clinical information obtained during index hospitalization, including ECG, serial laboratory measurements, monitoring of vital signs in the perioperative and intraoperative period, as well as cardiac imaging. In cases of disagreement between the two reviewers, consensus was sought and found by discussion with a third reviewer. PMI were hierarchically classified into: Extra-cardiac if caused by a primarily extra-cardiac disease such as severe sepsis, stroke, pulmonary embolism, or cardiac trauma; Cardiac if treated as T1MI, or caused by tachyarrhythmia, acute heart failure (AHF) or as PMI of unknown origin if there was absence of all previously mentioned causes.

**Treated as Type I myocardial infarction** was adjudicated when there was a need for an intervention or bypass surgery after coronary angiography due to presumed plaque rupture or a stenosis. In cases that there were contraindications or patient’s refusal to go to coronary angiography despite high suspicion of type I (ST-elevation, pronounced ST-depression, typical angina pectoris, strongly elevated cardiac troponin), an adjudication towards type I was done without coronary angiographic evidence.

**Tachyarrhythmia** was adjudicated if there was evidence of a tachyarrhythmia on ECG with ≥120 beats per minute.

**Acute heart failure** was adjudicated in cases with signs of congestion (dyspnea, peripheral oedema, pleural effusion, rales), use of iv-diuretics improving symptoms, or elevated natriuretic peptides (BNP/NT-proBNP).

**Extra-cardiac PMI** was adjudicated if there was postoperative evidence of septic shock, severe sepsis^8^ or an uncontrolled infection in combination with criteria of severe immune response syndrome without evidence of bacteria or fungi in blood culture; pulmonary embolism; cardiac trauma (during surgery, e.g. thoracic or vascular surgery) and other rare reasons could be added as “other” with free text.

## STROBE Statement—Checklist of items that should be included in reports of *cohort studies(8)*

|  | Item No | Recommendation | Page No |
| --- | --- | --- | --- |
| **Title and abstract** | 1 | (*a*) Indicate the study’s design with a commonly used term in the title or the abstract | 1 |
|  |  | (*b*) Provide in the abstract an informative and balanced summary of what was done and what was found | 2 |
| Introduction | | | |
| Background/rationale | 2 | Explain the scientific background and rationale for the investigation being reported | 3 |
| Objectives | 3 | State specific objectives, including any prespecified hypotheses | 3 |
| Methods | | | |
| Study design | 4 | Present key elements of study design early in the paper | 4 |
| Setting | 5 | Describe the setting, locations, and relevant dates, including periods of recruitment, exposure, follow-up, and data collection | 4-8 |
| Participants | 6 | (*a*) Give the eligibility criteria, and the sources and methods of selection of participants. Describe methods of follow-up | 4-8 |
|  |  | (*b*) For matched studies, give matching criteria and number of exposed and unexposed |  |
| Variables | 7 | Clearly define all outcomes, exposures, predictors, potential confounders, and effect modifiers. Give diagnostic criteria, if applicable | 4-8 |
| Data sources/ measurement | 8* | For each variable of interest, give sources of data and details of methods of assessment (measurement). Describe comparability of assessment methods if there is more than one group | 4-8 |
| Bias | 9 | Describe any efforts to address potential sources of bias | 4-8 |
| Study size | 10 | Explain how the study size was arrived at | 4-8 |
| Quantitative variables | 11 | Explain how quantitative variables were handled in the analyses. If applicable, describe which groupings were chosen and why | - |
| Statistical methods | 12 | (*a*) Describe all statistical methods, including those used to control for confounding | 7-8 |
|  |  | (*b*) Describe any methods used to examine subgroups and interactions | - |
|  |  | (*c*) Explain how missing data were addressed | - |
|  |  | (*d*) If applicable, explain how loss to follow-up was addressed | - |
|  |  | (*e*) Describe any sensitivity analyses | - |
| Results | | |  |
| Participants | 13* | (a) Report numbers of individuals at each stage of study—eg numbers potentially eligible, examined for eligibility, confirmed eligible, included in the study, completing follow-up, and analysed | 8-9 |
|  |  | (b) Give reasons for non-participation at each stage | - |
|  |  | (c) Consider use of a flow diagram | figure 1 |
| Descriptive data | 14* | (a) Give characteristics of study participants (eg demographic, clinical, social) and information on exposures and potential confounders | 8-9 |
|  |  | (b) Indicate number of participants with missing data for each variable of interest | - |
|  |  | (c) Summarise follow-up time (eg, average and total amount) | 8-9 |
| Outcome data | 15* | Report numbers of outcome events or summary measures over time | 8-9 |

| Main results | 16 | (*a*) Give unadjusted estimates and, if applicable, confounder-adjusted estimates and their precision (eg, 95% confidence interval). Make clear which confounders were adjusted for and why they were included | 8-9 |
| --- | --- | --- | --- |
|  |  | (*b*) Report category boundaries when continuous variables were categorized | 8-9 |
|  |  | (*c*) If relevant, consider translating estimates of relative risk into absolute risk for a meaningful time period | - |
| Other analyses | 17 | Report other analyses done—eg analyses of subgroups and interactions, and sensitivity analyses | 8-9 |
| Discussion | | | |
| Key results | 18 | Summarise key results with reference to study objectives | 10-12 |
| Limitations | 19 | Discuss limitations of the study, taking into account sources of potential bias or imprecision. Discuss both direction and magnitude of any potential bias | 12 |
| Interpretation | 20 | Give a cautious overall interpretation of results considering objectives, limitations, multiplicity of analyses, results from similar studies, and other relevant evidence | 12 |
| Generalisability | 21 | Discuss the generalisability (external validity) of the study results | 10-12 |
| Other information | | | |
| Funding | 22 | Give the source of funding and the role of the funders for the present study and, if applicable, for the original study on which the present article is based | 18 |

## Supplemental Figures


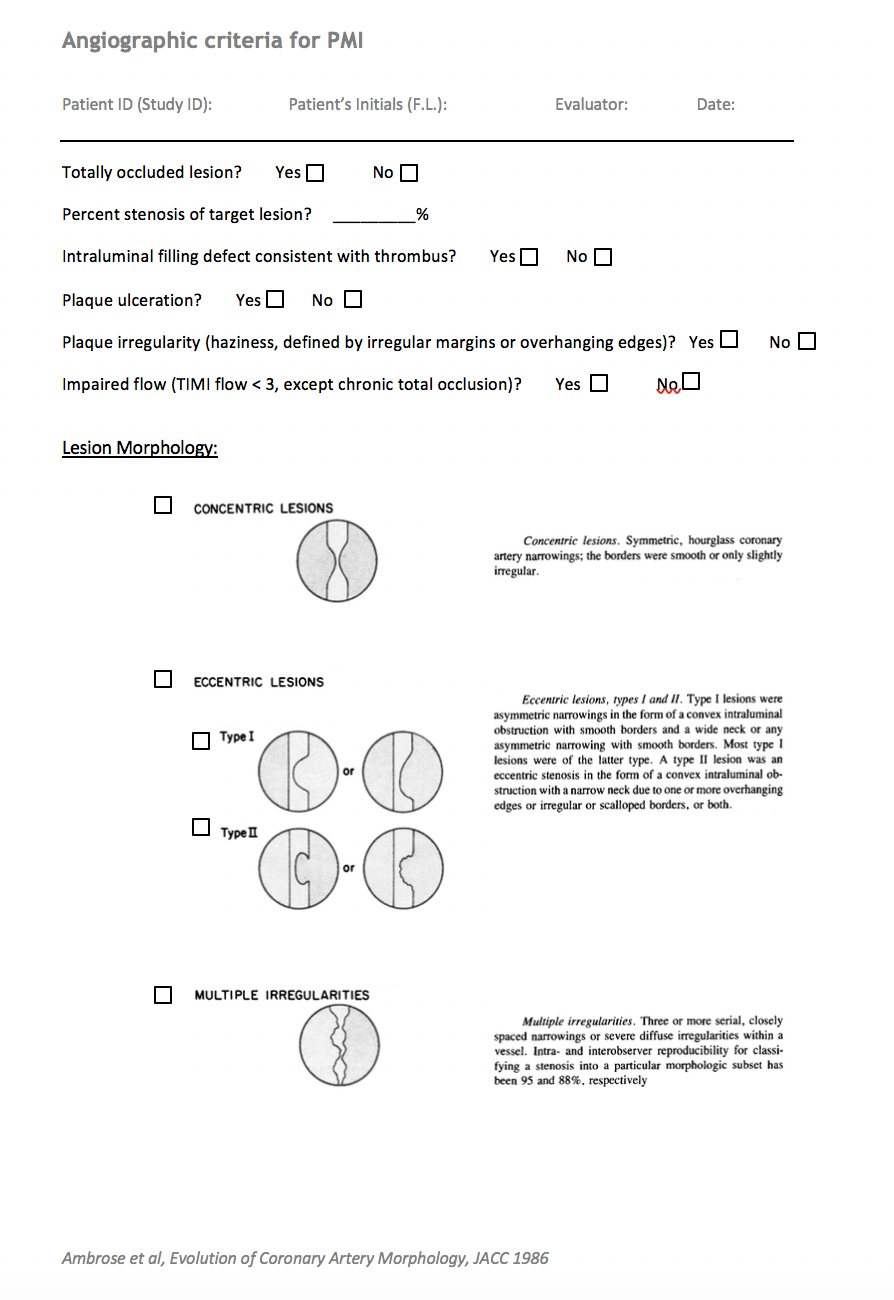


***Figure 1:*** Case report form for the angiographic findings (9,10)


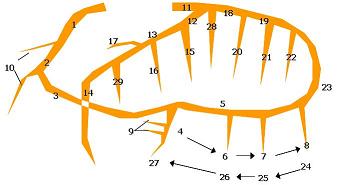


***Figure 2:*** Orientation chart for the description of the affected vessel (11)

1.Proximal right coronary artery conduit segment, 2.Mid-right coronary artery conduit segment, 3.Distal right coronary artery conduit segment, 4.Right posterior descending artery segment, 5.Right posterior atrioventricular segment, 6.First right posterolateral segment, 7.Second right posterolateral segment, 8.Third right posterolateral segment, 9.Posterior descending septal perforators segment, 10.Acute marginal segment, 11.Left main coronary artery segment, 12.Proximal LAD artery segment, 13.Mid-LAD artery segment, 14.Distal LAD artery segment, 15.First diagonal branch segment, 16.Second diagonal branch segment, 17.LAD septal perforator segments, 18.Proximal circumflex artery segment, 19.Mid-circumflex artery segment, 20.First obtuse marginal branch segment, 21.Second obtuse marginal branch segment, 22.Third obtuse marginal branch segment, 23.Circumflex artery AV groove continuation segment, 24.First left posterolateral branch segment, 25.Second left posterolateral branch segment, 26.Third posterolateral descending artery segment, 27.Left posterior descending artery segment, 28.Ramus intermedius segment, 29.Third diagonal branch segment

# Supplemental References

1. Vascular Events In Noncardiac Surgery Patients Cohort Evaluation Study I, Devereaux PJ, Chan MT et al. Association between postoperative troponin levels and 30-day mortality among patients undergoing noncardiac surgery. JAMA 2012;307:2295-304.

2. Kavsak PA, MacRae AR, Yerna MJ, Jaffe AS. Analytic and clinical utility of a next-generation, highly sensitive cardiac troponin I assay for early detection of myocardial injury. Clin Chem 2009;55:573-7.

3. Weber M, Luchner A, Seeberger M et al. Incremental value of high-sensitive troponin T in addition to the revised cardiac index for peri-operative risk stratification in non-cardiac surgery. Eur Heart J 2013;34:853-62.

4. Irfan A, Reichlin T, Twerenbold R et al. Early diagnosis of myocardial infarction using absolute and relative changes in cardiac troponin concentrations. Am J Med 2013;126:781-788 e2.

5. Reichlin T, Irfan A, Twerenbold R et al. Utility of absolute and relative changes in cardiac troponin concentrations in the early diagnosis of acute myocardial infarction. Circulation 2011;124:136-45.

6. Thygesen K, Alpert JS, Jaffe AS et al. Fourth Universal Definition of Myocardial Infarction (2018). Circulation 2018;138:e618-e651.

7. Apple FS. A new season for cardiac troponin assays: it's time to keep a scorecard. Clin Chem 2009;55:1303-6.

8. von Elm E, Altman DG, Egger M et al. The Strengthening the Reporting of Observational Studies in Epidemiology (STROBE) statement: guidelines for reporting observational studies. Lancet 2007;370:1453-7.

9. Ambrose JA, Winters SL, Stern A et al. Angiographic morphology and the pathogenesis of unstable angina pectoris. J Am Coll Cardiol 1985;5:609-16.

10. Goldstein JA, Demetriou D, Grines CL, Pica M, Shoukfeh M, O'Neill WW. Multiple complex coronary plaques in patients with acute myocardial infarction. N Engl J Med 2000;343:915-22.

11. Scanlon PJ, Faxon DP, Audet AM et al. ACC/AHA guidelines for coronary angiography. A report of the American College of Cardiology/American Heart Association Task Force on practice guidelines (Committee on Coronary Angiography). Developed in collaboration with the Society for Cardiac Angiography and Interventions. J Am Coll Cardiol 1999;33:1756-824.
